# C-Reactive Protein +1444C/T Polymorphism Is Associated with the Susceptibility to Pulmonary Tuberculosis

**DOI:** 10.1155/2020/6634879

**Published:** 2020-12-19

**Authors:** Yuzhong Xu, Minggang Cheng, Xiong Wang

**Affiliations:** ^1^Department of Clinical Laboratory, Shenzhen Baoan Hospital, Southern Medical University, Shenzhen 518000, China; ^2^Department of Laboratory Medicine, Tongji Hospital, Tongji Medical College, Huazhong University of Science, Wuhan 430030, China

## Abstract

**Objective:**

The T allele of C-reactive protein (CRP) +1444C/T (rs1130864) polymorphism was associated with increased risk for some inflammatory conditions. The objective of the study was to explore the association between the CRP +1444C/T polymorphism with the susceptibility to pulmonary tuberculosis (PTB) in a Chinese population.

**Methods:**

This case-control study enrolled 480 PTB patients and 480 healthy controls. The CRP +1444C/T polymorphism was determined using Sanger sequencing. The odds ratio (OR) and 95% confidence interval (CI) were assessed to examine the strength of genetic correlation.

**Results:**

The genotype and allele frequencies of PTB patients differed from controls (CT vs. CC, OR = 1.924, 95% CI: 1.099-3.371, adjusted *P* value = 0.022; T vs. C, OR = 1.884, 95% CI: 1.085-3.273, adjusted *P* value = 0.024). Stratified analysis by sex found that PTB patients' genotype and allele frequencies differed from controls in the male subgroup but not the female subgroup.

**Conclusion:**

In conclusion, the minor T allele of CRP +1444C/T polymorphism was associated with increased PTB risk.

## 1. Introduction

Tuberculosis (TB) is an infectious disease caused by *Mycobacterium tuberculosis* (Mtb), which ranks among the top 10 causes of death worldwide. According to the global tuberculosis report 2019 from WHO (https://www.who.int/tb/publications/global_report/en/), about 10.0 million people fell ill with TB in 2018, and 1.7 billion people were latent TB infection (LTBI). Early and accurate diagnosis of TB is critical for achieving the goal of global tuberculosis control [1]. Most of the Mtb infections are LTBI, which is asymptomatic. When the host immune system weakens, the Mtb becomes active and causes active TB with clinical symptoms. The development of TB is affected by the characteristics of Mtb, the host immune responses, and the genetic susceptibility [2].

Plasma C-reactive protein (CRP) is an acute-phase reactant produced by hepatocytes after an IL-6 stimulus and plays an essential role in chronic and acute inflammation. Plasma CRP is a useful nonspecific biomarker for the assessment of disease activity in inflammatory conditions, including TB. Serum CRP was increased in TB, being highest in advanced disease. This level fell with the anti-TB drug treatment and also correlated with the clinical response [3, 4]. The CRP +1444C/T (rs1130864) polymorphism was located in the 3′ untranslated region (3′-UTR) of the CRP gene. The T allele carriers had higher CRP serum concentrations [5]. This polymorphism has been found to be associated with the susceptibility to several inflammatory conditions, such as systemic lupus erythematosus (SLE) and cardiovascular disease (CVD) [6, 7]. However, the association between the CRP +1444C/T (rs1130864) polymorphism and the susceptibility to pulmonary TB (PTB) is not yet studied.

The objective of the study was to explore the association between the CRP +1444C/T polymorphism and the susceptibility to PTB in a Chinese population including 480 PTB patients and 480 healthy controls.

## 2. Materials and Methods

### 2.1. Subjects

480 PTB patients were enrolled from Shenzhen Baoan Hospital, Southern Medical University from January 2018 to January 2020. PTB was diagnosed by chest X-ray radiography and clinical symptoms including (fever, hemoptysis, weight loss, dyspnea, chest pain, and night sweats), combined with positive result of culture, Mtb-specific PCR, T-SPOT.TB assay, or Xpert MTB/RIF. Patients with HIV infection, immunodeficiency disease, diabetes mellitus, tumor, or other lung diseases were excluded. This study was approved by the ethics committee of Shenzhen Baoan Hospital. Informed consent was obtained from all subjects.

### 2.2. Genotyping of the CRP +1444C/T Polymorphism

200 *μ*l peripheral blood was collected, and genomic DNA was extracted using the GeneRotex 96 Nucleic Acid Extraction workstation (TIANLONG, Xi'an, China). The following primers were used: forward, 5′-ATATTAATAAGGAGCTCGTTAACTATGCTGGGACA-3′ and reverse, 5′-TTCTCAGCTCTTGCCTTATGAG-3′ according to a previous study [8]. The PCR fragments were sequenced using ABI 3500 Dx (ABI, San Diego, CA, USA), and the results were blasted on NCBI.

### 2.3. Statistical Analyses

All data were presented as mean ± SD and analyzed using SPSS 18.0 (SPSS Inc., Chicago, Illinois, USA). Differences of age between two groups were analyzed using unpaired Student's *t*-test. Chi-squared (*χ*^2^) test was applied to assess the Hardy-Weinberg equilibrium (HWE) in controls. Odds ratios (OR) and 95% confidence intervals (CIs) were calculated using binary logistic regression analysis, adjusted by age combined with/without sex. *P* value < 0.05 was considered statistically significant.

## 3. Results

### 3.1. Demographic and Clinical Characteristics of Subjects

480 PTB patients (mean age: 39.81 ± 12.65 years) including 330 males and 150 females and matched 480 healthy controls (mean age: 39.88 ± 14.61 years) including 330 males and 150 females were enrolled. No significant difference was found in either age or male ratio between cases and controls. Detailed clinical characteristics were listed in Table 1.

### 3.2. Association between CRP +1444C/T Polymorphism and the Susceptibility to PTB

The controls followed the HWE principle (*P* = 0.641), suggesting representative control subjects. The genotype distribution of CRP +1444C/T polymorphism in cases was significantly different from controls (CT vs. CC, OR = 1.924, 95% CI: 1.099-3.371, adjusted *P* value = 0.022). Stratified analysis by sex found that the genotype distribution of CRP +1444C/T polymorphism in cases was significantly different from male controls (CT vs. CC, OR = 2.178, 95% CI: 1.127-4.208, adjusted *P* value = 0.021) but not females (Table 2).

The allele frequencies of CRP +1444C/T polymorphism was significantly different from controls (T vs. C, OR = 1.884, 95% CI: 1.085-3.273, adjusted *P* value = 0.024). Stratified analysis by sex found that the genotype distribution of CRP +1444C/T polymorphism in cases was significantly different from male controls (T vs. C, OR = 2.120, 95% CI: 1.109-4.052, adjusted *P* value = 0.023) but not females (Table 3).

## 4. Discussion

Detection of TB is still challenging, although great advances have been achieved in new diagnostic methods [1]. Difficulty exists in detecting several cases such as acid-fast cacillus (AFB) smear-negative sputum, adolescent TB, and reactivation of LTBI. The diagnosis of adolescent TB is challenging as adolescent TB is paucibacillary with nonspecific symptoms. In the early stage, up to 50% of adolescent TB can be asymptomatic [9]. Inexpensive and simple diagnostic methods have not been found yet. Several biomarkers have been identified for diagnosis and differentiation between active TB and LTBI [10, 11].

CRP is an acute-phase reactant with a short half-life and has been used as one of the most frequent ways of monitoring inflammation [12]. CRP participates in the immune response by binding to its main ligand phosphorylcholine or intracellular ligands, such as chromatin, ribonucleoprotein, and histones on dying or dead cells, activates the complement pathway and Fc-receptor-mediated effectors pathways, and promotes phagocytosis [13–15]. CRP, soluble intercellular adhesion molecule-1 (sICAM1), procalcitonin, and neopterin are serum biomarkers which exhibit macrophage activation in TB [4]. Serum CRP level was increased in TB patients, especially in those patients with AFB smear-positive sputum. Serum CRP level was correlated with severe lung tissue damage [16]. CRP level decreased to a normal level after anti-TB treatment, indicating an effective therapeutic response [4].

The gene coding for CRP is located at 1q.23.2. Some functional polymorphisms within the CRP gene have been found to affect serum CRP levels, including the CRP +1444C/T (rs1130864) polymorphism, rs3091244, rs3093077, and rs11265265 [5, 17–20]. Associations between the CRP +1444C/T polymorphism and the susceptibility to several diseases have been widely studied. The CRP +1444C/T polymorphism was associated with SLE risk, and patients carrying the T allele presented higher CRP levels [6]. The CRP +1444C/T polymorphism was significantly associated with increased posttraumatic stress disorder (PTSD) symptoms and increased CRP level [21]. The minor allele of CRP +1444C/T polymorphism was associated with decreased risk of depression in women aged at least 65 years. CRP gene variant was also associated with serum levels in a gender-specific manner. The CRP +1444C/T polymorphism may influence circulating CRP level [22]. In the current study, we found that the T allele of CRP +1444C/T polymorphism was associated with increased PTB risk, especially in males. In the included 480 PTB patients, only 150 patients were female. The limited sample size of female may be the reason why this association was not found in female.

Some limitations exist in our study. First, the sample size was small, and only 150 female cases were enrolled. A larger sample size in female population deserves further study of the association between CRP +1444C/T polymorphism and PTB risk in female. Second, the information of the serum CRP level was lacking. Third, the *P* value was only adjusted by age and/or sex.

In conclusion, our data indicate that the minor T allele of CRP +1444C/T polymorphism was associated with increased PTB risk in a Chinese population, especially in males.

## Figures and Tables

**Table 1:** Characteristics of the study population.

Variable	Case	Control	*P* value
Number	480	480	
Sex			
Male	330 (68.75%)	330	
Female	150 (31.25%)	150	
Age	39.81 ± 12.65	39.88 ± 14.61	0.938
Male	40.22 ± 12.48	40.32 ± 15.12	0.926
Female	38.93 ± 13.03	38.93 ± 13.41	1.000

**Table 2:** Genotype distribution of CRP +1444C/T polymorphism in all subjects.

Number	Case	Control	OR	Adjusted *P* value
	480	480		
Genotype				
CC	443 (92.29%)	460 (95.83%)	1	
CT	37 (7.71%)	20 (4.17%)	1.924 (1.099-3.371)	0.022
Male number	330	330		
CC	301 (91.21%)	316 (95.76%)	1	
CT	29 (8.79%)	14 (4.24%)	2.178 (1.127-4.208)	0.021
Female number	150	150		
CC	142 (94.67%)	144 (96%)	1	
CT	8 (5.33%)	6 (4%)	1.352 (0.457-3.999)	0.585

The *P* value was adjusted by age combined with/without sex.

**Table 3:** The allele frequencies of CRP +1444C/T polymorphism in all subjects.

Model	Allele	Case	Control	OR (95% CI)^∗^	Adjusted *P* value^∗^
Overall	C	923 (96.15%)	940 (97.92%)	1	
	T	37 (3.85%)	20 (2.08%)	1.884 (1.085-3.273)	0.024
Male	C	631 (95.61%)	646 (97.88%)	1	
	T	29 (4.39%)	14 (2.12%)	2.120 (1.109-4.052)	0.023
Female	C	292 (97.33%)	294 (98%)	1	
	T	8 (2.67%)	6 (2%)	1.343 (0.460-3.918)	0.590

The *P* value was adjusted by age combined with/without sex.

## Data Availability

The datasets used and analyzed during the current study are available from the corresponding author on reasonable request.
